# A Community-Based Partnership to Successfully Implement and Maintain a Breast Health Navigation Program

**DOI:** 10.1007/s10900-015-0051-z

**Published:** 2015-06-16

**Authors:** Bettina F. Drake, Shivon Tannan, Victoria V. Anwuri, Sherrill Jackson, Mark Sanford, Jennifer Tappenden, Melody S. Goodman, Graham A. Colditz

**Affiliations:** Division of Public Health Sciences, Department of Surgery, Washington University School of Medicine, 600 S. Taylor Ave., Campus Box 8100, St. Louis, MO 63110 USA; Alvin J. Siteman Cancer Center, St. Louis, MO USA; Institute of Public Health, Washington University, St. Louis, MO USA; Betty Jean Kerr People’s Health Centers, St. Louis, MO USA

**Keywords:** Breast cancer, Mammography, Screening, Patient navigation, Disparities

## Abstract

Breast cancer screening combined with follow-up and treatment reduces breast cancer mortality. However, in the study clinic, only 12 % of eligible women ≥40 years received a mammogram in the previous year. The objective of this project was to implement patient navigation, in our partner health clinic to (1) identify women overdue for a mammogram; and (2) increase mammography utilization in this population over a 2-year period. Women overdue for a mammogram were identified. One patient navigator made navigation attempts over a 2-year period (2009–2011). Navigation included working around systems- and individual-level barriers to receive a mammogram as well as the appropriate follow-up post screening. Women were contacted up to three times to initiate navigation. The proportion of women navigated and who received a mammogram during the study period were compared to women who did not receive a mammogram using Chi square tests for categorical variables and *t* tests for continuous variables with an α = 0.05. Barriers to previous mammography were also assessed. With 94.8 % of eligible women navigated and 94 % of these women completing mammography, the implementation project reached 89 % of the target population. This project was a successful implementation of an evidence-based patient navigation program that continues to provide significant impact in a high-need area. Cost was the most commonly cite barrier to mammography. Increasing awareness of resources in the community for mammography and follow-up care remains a necessary adjunct to removing structural and financial barriers to accessing preventive services.

## Introduction

Breast cancer screening combined with appropriate follow-up and treatment reduces breast cancer mortality [1]. Studies suggest that many women begin regular mammography screening later than recommended, do not have mammograms at recommended intervals, and do not receive appropriate and timely follow-up [2–4]. This is especially true for women living in areas with minimal access to medical facilities. Patient navigation programs have emerged as a potential solution for improving cancer care delivery [5, 6] and a growing number of studies documenting the promise of navigation have resulted in its widespread adoption [7–9]. Navigation contributes to the early detection of breast cancer by reducing barriers such as income, insurance status, access to care and facility to navigate through care among women to obtain breast cancer screening [10, 11].

The first patient navigation program was started in New York to increase the delivery of mammography screening to Black women who were too often presenting with advanced cancer as a result of a lack of screening [12]. The process of patient navigation facilitates access to quality medical care by identifying barriers to care and bridging gaps in care through culturally sensitive coordination. Patient navigators are resources for patients and providers and may assist with all phases of access, including primary care prevention, screening and follow-up care, cancer treatment, and survivorship care [13].

Advanced breast cancer diagnosis is more likely to occur in areas with higher proportions of minority racial/ethnic groups or low median household income, which tend to correlate with service access [14, 15]. This association extends to screening, in which areas with higher poverty and lower mammography usage are more likely located in high breast cancer incidence clusters [16]. The Program for the Elimination of Cancer Disparities (PECaD), a NCI-funded Community Networks Program at Washington University in St. Louis and Siteman Cancer Center, works with community partners within its Breast Cancer Community Partnership to advance the elimination of breast cancer disparities and identify gaps and barriers to cancer screening and treatment in local underserved communities. Previous work [17, 18] on delays in mammography screening to diagnosis in the region has revealed a median time from first sign to definitive diagnosis of 93 days [18]. Both system- and patient-specific factors were found to be associated with delayed diagnosis/treatment in breast patients referred to an academic cancer center.

One of the partners of the Breast Cancer Community Partnership represents one of the local FQHCs, Betty Jean Kerr People’s Health Centers (PHC). PHC one of the few federally-qualified healthcare centers (FQHC) in the region that had capacity to conduct mammograms. Through this project, PHC added mammography services to its second of three clinics in North St. Louis (the study clinic). In 2009, only 12 % of eligible women served by the study clinic, 40 years and older received a mammogram in the previous year. In the same year, 60 % of women 40 years and older were uninsured or underinsured and 87 % of patients served by all three PHC clinics are African American. PHC provides the third largest primary care volume to the uninsured and Medicaid patients in the St. Louis region [19].

Together, community partners from PHC and academics from PECaD applied and received American Recovery and Reinvestment Act (ARRA) funds to implement an evidence-based program to increase mammography utilization in an underserved area with an identified late-stage breast cancer cluster [20]. When developing our ARRA project, PHC’s largest location (Delmar site) had a patient navigator that served as a case manager to provide patient advocacy within and across provider systems, encouragement and support for breast cancer prevention, education and screening. Through the ARRA project we funded a second patient navigator to serve at the second largest and fastest growing PHC location (North St. Louis county site), which is located closer to the late-stage breast cancer diagnosis cluster [20], and is more accessible to the community and target population respective to reducing breast cancer health disparities.

The study clinic is one of three clinics within PHCs, which did not provide mammography services or navigation at study baseline. The purpose of this project was to implement an evidence-based program, patient navigation, in a high-need area to (1) identify women due or overdue for a mammogram; and (2) increase mammography utilization in this population over a 2-year period.

## Methods

Mammography services did not exist at the North St. Louis county clinic prior to this study period. Women due or overdue for a mammogram at this clinic were identified. One patient navigator made navigation attempts over a 2-year period (2009–2011). In addition, barriers or reasons for being due or overdue for a mammogram were assessed. Analyses were conducted in 2013.

### Study Setting

In an analysis of Missouri breast cancer data, a geographic cluster of elevated, first primary diagnosis of late-stage breast cancer was identified in an eight zip code section of St. Louis referred to as North St. Louis City [20]. In St. Louis, the majority of the African-American population is located in the northern area of the city, where this late-stage cluster is located [21]. Among females in this cluster, 36.6 % are African-American and over 40 years of age; 29.8 % of all African-American males and females have incomes below the poverty level [22].

### Study Population

Women were identified as due or overdue for a mammogram by the patient navigator for navigation based on age and time since last mammogram according to study clinic medical records (N = 792). Mammography guidelines from the American Cancer Society (ACS) were used. Women age 40 year and over should have a mammogram and clinical breast exam by a health professional every year [23]. 792 women were identified as being due or overdue for a mammogram and therefore were in need of navigation. The Washington University IRB approved this study.

### Inclusion Criteria

Patient navigation was implemented in the North St. Louis county location of PHC (study clinic) and only patients of this location of PHC were included in this study.

### Patient Navigation

The navigator used two strategies to further identify women: (1) the navigator identified women were due or overdue for a mammogram based on the length of time since their last recorded mammogram; and (2) each day, the navigator targeted women attending the study clinic for reasons other than mammography who were overdue for a mammogram and enrolled them into the study to be navigated through the breast cancer screening process.

The responsibilities of the patient navigator at the North St. Louis county location included, but were not limited to, developing collaborative relationships with cancer service and treatment providers to navigate women in the system that are eligible for and due/overdue for a mammogram, providing face-to-face, telephone and mail-based support to connect women to appropriate screening, diagnostic and treatment services, assisting women through the initial and follow-up visit process for mammogram related care, locating women that have lost contact with the system, working with women to identify barriers to mammography, and providing assistance to patients as needed to encourage appointment attendance (e.g. help arranging public transportation or finding childcare resources, etc.).

Women were contacted up to three times to initiate navigation. Patients with three unsuccessful navigation attempts were scheduled to be recontacted the following year.

### Barriers

Data was collected from the navigated women on barriers that contributed to being overdue for her breast screening. During the initial contact, the navigator asked the women if any of the listed barriers prevented them from receiving a mammogram every year, according to ACS guidelines [23]. Barriers included: my doctor required a clinical breast exam prior to receiving a mammogram, I had a mammogram elsewhere, my language or culture was a barrier, I could not afford to receive a mammogram, or other reason. Women could choose more than one barrier.

### Data Analysis

Descriptive statistics were used to analyze baseline sociodemographic characteristics between women who received a mammogram and women who did not receive a mammogram. In an analysis of Missouri breast cancer data, a geographic cluster of elevated diagnosis of late-stage breast cancer was identified [20]. The geographic location of women living in or out of this late-stage cluster was recorded. The proportion of mammography utilization for the North St. Louis county location over the study period is compared to mammography utilization for all PHC locations. Barriers to receiving a mammogram were reported as well as the number of women who reported multiple barriers. χ^2^ and corresponding *p* values are reported to assess statistical significance. The Chi squared values are the test statistic for a two sample difference of proportion test, with the two samples being whether the participant had a mammogram or not. When the count of participants in a particular category fell below six, a Fisher Exact Test was used in place of the difference of proportion test. The exact test was used in these cases because the difference of proportion test, which rests on asymptotic arguments, performs poorly with low counts. SAS 9.2 was used for analyses.

## Results

Table 1 shows the demographics of women (N = 792) at the North St. Louis county location that were identified as needing navigation by the fact that they were due or overdue for a mammogram within the study period. Among the women identified as needing navigation, 89.3 % were African-American, 99 % were non-Hispanic; 37.1 % were unemployed; and 57 % were uninsured. The majority of the women needing navigation had a high school degree or less education (56.4 %) and an income less than $15,000 (55.3 %). About 1/3 (30.4 %) of the patients identified as needing navigation were residents of a late-stage cluster [20] within the catchment area of the health center. There is no difference by demographic variables between women who received a mammogram after navigation and women who did not receive a mammogram after navigation attempts. 89 % of the total women identified as needing navigation were African-American and there were no significant racial differences between women who received a mammogram and those who did not receive a mammogram (*p* = 0.911) and the population was largely non-Hispanic ethnicity (0.6 %). More than half of the population had a high school degree or below (56.4 %). 41.8 % of women identified as needing navigation were 40–49 years old and 35.5 % were 50–59 years old. 20 % of women due or overdue for a mammogram were 60 years of age or over. Over one-third of the population was unemployed (37.1 %) and over half made an annual income less than $15,000 (55.3 %). 57 % of the total population was uninsured and 17.9 % had private insurance. A slightly higher percentage of women were navigated to receive a mammogram (58.2 %) compared to women who did not receive a mammogram (55.0 %). 31 % of women who received a mammogram lived in the late-stage cluster.

**Table 1:** Patient demographics from 2009 to 2012 (N = 792)

	Needs navigation	Navigated women	Received mammogram	No mammogram	χ^2^ (*p* value)*
	Total N (%)	n (%)	n (%)	n (%)	
Race					
Black/African American	707 (89.3)	674 (89.8)	577 (89.9)	97 (89.0)	0.012 (0.911)
White	72 (9.1)	66 (8.8)	55 (8.6)	11 (10.1)	0.113 (0.736)
Other	12 (1.5)	10 (1.3)	9 (1.4))	1 (0.9)	(0.999)^c^
Refused	1 (0.1)	1 (0.1)	1 (0.1)	0 (0.0)	(0.999)^c^
Ethnicity					
Hispanic/Latino	5 (0.6)	5 (.67)	5 (0.7)	0 (0.0)	(0.999)^c^
Not Hispanic/Latino	784 (99.0)	743 (98.9)	634 (98.8)	109 (100.0)	0.445 (<0.504)
Refused	3 (0.4)	3 (0.4)	3 (0.5)	0 (0.0)	(0.999)^c^
Education					
Less than high school	77 (9.7)	69 (9.2)	58 (9.0)	11 (10.1)	0.030 (<0.861)
High school degree	447 (56.4)	431 (57.4)	367 (57.2)	64 (58.7)	0.039 (<0.843)
Some college/Associates degree	171 (21.6)	159 (21.2)	139 (21.7)	20 (18.3)	0.427 (<0.513)
Bachelors degree	54 (6.8)	51 (6.8)	44 (6.8)	7 (6.4)	0 (0.999)
Masters degree	5 (0.6)	4 (.53)	2 (0.3)	2 (1.8)	(0.102)^c^
Refused	38 (4.8)	37 (4.9)	32 (5.0)	5 (4.6)	0 (0.1)
Age in years					
<40	20 (2.5)	13 (1.7)	30 (4.6)	0 (0.0)	(0.014)^c^
40–49	331 (41.8)	299 (39.8)	261 (39.6)	42 (38.5)	0.011 (0.915)
50–59	281 (35.5)	259 (34.5)	215 (32.6)	46 (42.2)	3.408 (0.064)
60–69	144 (18.2)	137 (18.2)	118 (17.9)	20 (18.3)	0 (0.999)
70–89	16 (2.0)	16 (2.1)	15 (2.3)	1 (0.9)	(0.713)^c^
Refused		27 (3.6)	20 (3.0)		
Employment status					
Disabled	32 (4.0)	31 (4.1)	27 (4.2)	4 (3.7)	(0.999)^c^
Retired	46 (5.8)	46 (6.1)	35 (5.4)	11 (10.1)	2.728 (0.098)
Unemployed	294 (37.1)	283 (37.7)	238 (37.1)	45 (41.3)	0.536 (0.464)
Part time	74 (9.3)	68 (9.1)	57 (8.9)	11 (10.1)	0.051 (0.819)
Full time	313 (39.5)	291 (38.7)	260 (40.5)	31 (28.4)	5.211 (0.022)
Refused	33 (4.2)	32 (4.3)	25 (3.9)	7 (6.4)	0.905 (0.341)
Income					
<$15,000	438 (55.3)	419 (55.8)	351 (54.7)	68 (62.4)	1.945 (0.163)
$15,001–$25,000	198 (25.0)	184 (24.5)	163 (25.4)	21 (19.3)	1.572 (0.209)
$25,001–$35,000	82 (10.4)	76 (10.1)	64 (10.0)	12 (11.0)	0.026 (0.871)
$35,001–$45,000	29 (3.7)	28 (3.7)	26 (4.0)	2 (1.8)	(0.41)^c^
>45,000	12 (1.5)	12 (1.6)	11 (1.7)	1 (0.9)	(0.1)^c^
Refused	33 (4.2)	32 (4.3)	27 (4.2)	5 (4.6)	(0.798)^c^
Insurance status^a^					
Uninsured	480 (57.0)	480 (57.0)	420 (58.2)	60 (55.0)	5.590 (0.018)
Medicaid	104 (12.4)	104 (12.4)	94 (13.0)	10 (9.2)	0.005 (0.938)
Medicare	70 (8.3)	70 (8.3)	67 (9.3)	3 (2.8)	(0.098)^c^
Private^b^	151 (17.9)	151 (17.9)	141 (19.5)	10 (9.2)	2.264 (0.132)
Unknown	37 (4.4)	37 (4.4)		26 (23.8)	
Late-stage cluster					
Yes	241 (30.4)	227 (30.2)	199 (31)	28 (25.7)	
No	551 (69.6)	524 (69.8)	443 (69)	81 (74.3)	

* χ^2^ and corresponding *p* values compare differences between women who received a mammogram and women who did not receive a mammogram

^a^Non-mutually-exclusive data. Insurance status was recorded at each navigation episode (n = 842)

^b^Includes Department of Veterans Affairs insurance

^c^Fisher exact test, H_A_: OR ≠ 1

Among the women identified as needing navigation (N = 792) by the fact that they were due or overdue for a mammogram within the study period, 94.8 % (n = 751) received navigation services (see Table 2). Of the women who were navigated, 94.5 % (n = 710) got a mammogram during the study period. This includes 55 women who received a repeat mammogram in the second year of study. Therefore, 655 (87.2 %) individual women received a mammogram. With 94.8 % of eligible women navigated and 94 % of these women completing mammography, the implementation project reached 89 % of the target population. Only 14.5 % (n = 109) of women who received navigation did not receive a mammogram by the end of the 2-year study period. Navigation continued post-study as part of regular clinical care.

**Table 2:** North St. Louis Clinic navigation outcomes over 2 years

Navigation outcomes	N (%)
Women identified as due/overdue for mammogram	792
Women that received navigation	751 (94.8)
Mammograms received^a^	710^b^ (94.5)

^a^55 of the 710 mammograms received were repeat mammograms in year 2. 655 (87.2 %) unique women received mammography

^b^321 mammograms in year 1; 388 mammograms in year 2; 1 mammogram in year 3

Table 3 shows the age-eligible patient-population and mammography utilization during the study time period. The PHC study clinic had records of 906 female patients who were 40 years or older at baseline. Only 12 % of age-eligible women had received a mammogram at a PHC clinic other than the study site. After 1 year of implementing mammography services as well as navigation 17.7 % of all women seen at the PHC study clinic received a mammogram. This number increased to 27.6 % in year 2 of the study. The navigation program implemented in the study clinic also improved mammography number for all three of PHCs clinics combined. At baseline, 11.8 % of eligible women in all three centers received a mammogram; however, 15.4 % of eligible women in all three centers were receiving mammograms at the end of year 2 of the study. Six months of post-study data shows promise for continued reach and adoption of navigation and mammography services to women due or overdue after the end of the study period.

**Table 3:** People’s Health Centers breast cancer screening population and utilization (2009–current)

	Baseline	Year 1	Year 2	Post-study
	1/1/09 thru 9/29/09^a^	9/30/09 thru 9/29/10	9/30/10 thru 9/29/11	9/30/11 thru current^b^
	N (%)	N (%)	N (%)	N (%)
*All PHC clinics (3)*				
Age (years)				
40+	5317	6869	7112	5003
40–49	1759 (33.1)	2514 (36.6)	2644 (37.2)	1782 (35.6)
50+	3558 (66.9)	4355 (63.4)	4468 (62.8)	3221 (64.4)
Women receiving mammogram	627 (11.8)	931 (13.5)	1093 (15.4)	605 (12.1)
*PHC study clinic*				
Age (years)				
40+	906	1325	1522	1334
40–49	325 (35.4)	508 (38.3)	648 (42.6)	516 (38.7)
50+	581 (64.1)	817 (61.7)	874 (57.4)	818 (61.3)
Women receiving mammogram	108 (12)	235 (17.7)	420 (27.6)	250 (18.7)

^a^Baseline numbers before navigation starts

^b^Current is March 9, 2012—six months of data

### Barriers

**Fig. 1:**
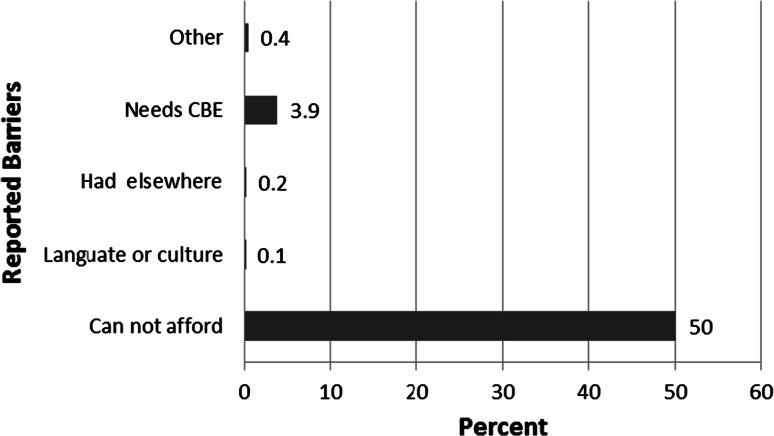
Reported barriers (374 women reported one barrier; 38 women reported two barriers; 2 women reported three barriers; and 1 woman reported four barriers) to receiving mammogram (n = 415). *CBE* clinical breast exam

415 women reported a barrier to being up-to-date with their breast cancer screening. For uninsured women without public coverage, out-of-pocket costs are a barrier to mammography [24]. In this study, 50 % of women navigated reported cost as a barrier to receiving a previous mammogram (see Fig. 1). At PHC, public and private funding sources can sometimes be used to offset out-of-pocket co-pays related to mammogram visits, however, all patients are assessed an initial co-pay at the on-set of a mammogram encounter series. The patient navigator offered co-pay coverage to women at the onset of mammogram related visits. There are several payment sources available in the region to cover health care costs related to mammography utilization including Medicare, Missouri Medicaid [25], Missouri Show Me Health Women [26] and Susan G. Komen for the Cure St. Louis Affiliate community-based grants [27]. In only 6.3 % of all navigation attempts women reported being able to pay the out-of-pocket co-pay required at the on-set of the mammogram encounter series. For women who did not qualify for one of the payment sources available in our region, the study provided co-pay vouchers. The study provided 106 vouchers (12.6 % of navigation attempts).

## Discussion

This project was a successful implementation of an evidence-based patient navigation program that continues to provide significant impact in a high-need area. This ARRA project was funded September 30, 2009 and navigation services began at the north location of PHC on October 1, 2009. The project provided funding to hire a patient navigator, a mammography technologist and a data coordination assistant for the study clinic. These positions/roles have been sustained post-ARRA funding by PHC. In addition, patient navigation for breast cancer screening has also been maintained at the study clinic. The navigator hired through this project has been integrated into the St. Louis Breast Health Navigator Work group which promotes sustainability, increased knowledge and sharing of best practices among more than 30 navigators from health centers and hospitals across the metropolitan region. This encourages sustainability of services at the study clinic, but also facilitates coordination of efforts when navigation women through the system from screening through treatment and follow-up. The sustainability of this effective program improves access to established preventive services [28], and increases community capacity.

In addition, this project filled a clear gap in geographic access to care. Without any special targeted efforts, 30 % of women navigated came from the late-stage cluster area. Targeted efforts may contribute to even greater success in reducing late-stage diagnoses in this area. Prior to the start of this project, all female patients of the study clinic received mammograms at a location central to the city. Women who utilized the study clinic as their medical home and lived near this location would have to commute via public transportation or personal vehicle 15–20 miles away (depending on route) to receive a mammogram. The addition of mammography services to the study clinic eliminates any transportation barriers that may exist. The reduction of structural barriers was recommended from the Guide to Community Preventive Services as an effective means to increase breast cancer screening [29].

During navigation, barriers to previous mammography use were assessed. Overwhelmingly, cost was cited as a barrier. Other commonly cited barriers in the literature such as fear, embarrassment, and concern about the procedure [30, 31], or difficulties with transportation and lack of knowledge and/or understanding of screening process or follow-up care directives [31–33] were not identified as barriers in this population. The real and perceived cost of screening and care is particularly prohibitive. Underinsured women, uninsured women and women with limited medical care resources are less likely to engage in screening and are more likely to delay care [20, 30, 31, 34, 35]. However, many women are unaware of the resources available to assist in the cost of screening. After the start of the study, the Guide to Community Preventive Services released a recommendation to reduce client out of pocket costs to increase breast cancer screening [29]. This is also in alignment with our study findings that future studies and programs should focus on increasing education about resources available for uninsured and underinsured women for screening—potentially through increased promotion of patient navigation.

This project was the result of collaborations through a community-based participatory research program. The funding mechanism (ARRA funds) focused on job creation and sustainability. Community partners at the study clinic brought the need of patient navigation and mammography services to the academic researchers. This is a strength of the study and is a strong contributing factor to the success of the program. In order to meet the needs of the funding mechanism, and to maximize the resources available to the study clinic and reduce delays in the initiation of navigation and mammography services, detailed summary reports were shared with all partners quarterly, rather than disseminating a dataset with individual patient records to partners outside of the study clinic. In addition, there was no control group receiving mammography without navigation; however our purpose was to introduce both components to maximize the benefits of breast screening by providing appropriate follow-up. There are also no data on repeat visits with or without navigation. While interrupted time series and similar outcome evaluation approaches are possible for implementation research the refinement and improvement of the navigation program should be conducted in the broader context of the overall network of providers. Despite the limitations, the data that is presented here presents a compelling argument for the success of an evidence-based program when implemented in a clinic that serves a high-need population.

There are a number of strengths to this project. An evidence-based program was successfully implemented in a high need area by a federally-qualified community health center that has a history of addressing the health needs of its surrounding communities and is well-positioned to continue to increase its health services and provide quality care. It has been sustained by the PHC and the barriers of access and cost have been reduced for a significant percentage of uninsured and underinsured patients.

Increasing awareness of resources in the community for mammography and follow-up care remains a necessary adjunct to removing structural and financial barriers to accessing preventive services. Patient navigation for breast cancer screening has the potential to reduce racial and geographic disparities in access to screening and in-turn improve breast cancer outcomes, when appropriately implemented in a high-need area. The financial benefits of medicaid expansion have the potential to increase use of preventive health services and reduce financial strain [36]. Increasing awareness of resources in the community for mammography and follow-up care remains a necessary adjunct to removing structural barriers to accessing preventive services.
